# The Transcription Repressor REST in Adult Neurons: Physiology, Pathology, and Diseases^1^,^2^,^3^

**DOI:** 10.1523/ENEURO.0010-15.2015

**Published:** 2015-07-10

**Authors:** Pietro Baldelli, Jacopo Meldolesi

**Affiliations:** 1 Department of Experimental Medicine, University of Genova, 16163 Genova, Italy; 2 Department of Neuroscience, San Raffaele Scientific Institute, 20132 Milano, Italy; 3 Department of Neuroscience and Brain Technologies, Istituto Italiano di Tecnologia, 16132 Genova, Italy; 4 Vita Salute San Raffaele University, 20132 Milano, Italy

**Keywords:** channels, excitotoxicity, mRNA splicing, neural genes, receptors, transcription repression

## Abstract

REST [RE1-silencing transcription factor (also called neuron-restrictive silencer factor)] is known to repress thousands of possible target genes, many of which are neuron specific. To date, REST repression has been investigated mostly in stem cells and differentiating neurons. Current evidence demonstrates its importance in adult neurons as well. Low levels of REST, which are acquired during differentiation, govern the expression of specific neuronal phenotypes. REST-dependent genes encode important targets, including transcription factors, transmitter release proteins, voltage-dependent and receptor channels, and signaling proteins. Additional neuronal properties depend on miRNAs expressed reciprocally to REST and on specific splicing factors. In adult neurons, REST levels are not always low. Increases occur during aging in healthy humans. Moreover, extensive evidence demonstrates that prolonged stimulation with various agents induces REST increases, which are associated with the repression of neuron-specific genes with appropriate, intermediate REST binding affinity. Whether neuronal increases in REST are protective or detrimental remains a subject of debate. Examples of CA1 hippocampal neuron protection upon depolarization, and of neurodegeneration upon glutamate treatment and hypoxia have been reported. REST participation in psychiatric and neurological diseases has been shown, especially in Alzheimer’s disease and Huntington’s disease, as well as epilepsy. Distinct, complex roles of the repressor in these different diseases have emerged. In conclusion, REST is certainly very important in a large number of conditions. We suggest that the conflicting results reported for the role of REST in physiology, pathology, and disease depend on its complex, direct, and indirect actions on many gene targets and on the diverse approaches used during the investigations.

## Significance Statement

Analysis of the role of REST (RE1-silencing transcription factor) in adult neurons, before and after stimulation and under pathological conditions, is presented for the first time, along with the available information about the role that the transcription repressor appears to play in diseases, especially in Alzheimer's disease and epilepsy. The development of these studies in the next few years is anticipated.

## General introduction

REST [RE1-silencing transcription factor (otherwise called neuron-restrictive silencer factor)], a zinc-finger transcription factor initially described as a nuclear negative regulator of differentiation (Chong et al., 1995; Schoenherr and Anderson, 1995), is now known to play a master role in neuronal cells (Ballas and Mandel, 2005; Gopalakrishnan, 2009). The effects of REST on its potential target genes depend on various factors, including the accessibility of the specific DNA binding sequences, the binding affinity, and the cooperation and competition with other transcription factors (Wu and Xie, 2006; Johnson et al., 2012; McClelland et al., 2014). Upon DNA binding, REST operates as a scaffold, assembling and positioning its operative complexes that include, among others, important enzymes such as histone deacetylases and the demethylase LSD1. These complexes are able to repress the transcription of large numbers of genes by modifying critical sites of their histones and DNA (Huang et al., 1999; Ballas and Mandel 2005; Ooi and Wood, 2007).

For quite some time, the REST target genes were described as containing one to five RE-1s (otherwise called neuron-restrictive silencer elements), the DNA sequences binding REST, in their promoter and other regulatory regions. Over a thousand RE-1-positive genes were reported, many of which were encoding for neuron-specific proteins (Bruce et al., 2004; Mortazavi et al., 2006; Johnson et al., 2007). Later on, further studies, performed in both neuronal and non-neuronal cell types, defined the RE-1 sequence as a subset of more numerous REST-binding motifs (Johnson et al., 2007, 2012; Otto et al., 2007; ENCODE Project Consortium, 2012). As a consequence, the number of potential REST targets increased considerably (several thousands). The present list includes genes encoding for RNAs of various types, not only mRNAs, but also microRNAs (miRNAs), short hairpin RNAs (shRNAs), and long noncoding RNAs (ncRNAs; Johnson et al., 2009; Rossbach, 2011; Volvert et al., 2014).

To date, the majority of REST studies have been conducted on embryonic and neural stem cells, and on neural cells during differentiation (Sun et al., 2005; Greenway et al., 2007; Johnson et al., 2008). In embryonic stem cells, high levels of REST (Jørgensen et al., 2009), working coordinately with other factors such as the canonical pluripotency factors Oct4, Sox2, and Nanog, were shown to operate in the repression of a large number of genes (Johnson et al., 2008). In neural stem cells that still exhibit high REST levels, the numerous genes repressed by REST coincide only in part with those of embryonic stem cells (Johnson et al., 2008). In addition to repression, REST has been shown to promote gene expression, which has been reported at all stages of cell differentiation (Ooi and Wood, 2007; Jobe et al., 2012). Such a dual role of REST is due to its ability to cross talk with other factors, including those governed by the Polycomb complexes, which enables it to participate in regulatory networks of transcription.

Neuronal differentiation soon reaches an advanced progenitor stage in which REST rapidly decreases to very low levels. This is due in part to the decreased control by two important regulators, β-catenin and HIPPI (HIP1 protein interactor; Willert et al., 2002; Nishihara et al., 2003; Datta and Bhattacharyya, 2011), that cooperate with the T-cell factor and SP1 transcription factors (Nishihara et al., 2003; Ravache et al., 2010). Moreover, over the course of differentiation SP1 is progressively replaced by its homolog SP3, with a further large decrease of transcription (Ravache et al., 2010). Concomitantly, a ubiquitinating enzyme, SCF-TRCP, which is specific for REST, undergoes rapid overexpression. An increase in the activity of this enzyme, which drives REST to proteolytic degradation, when accompanied by no change in the deubiquitinase enzyme HAUSP (Westbrook et al., 2008; Huang et al., 2011), greatly increases the turnover of REST.

**Figure 1:**
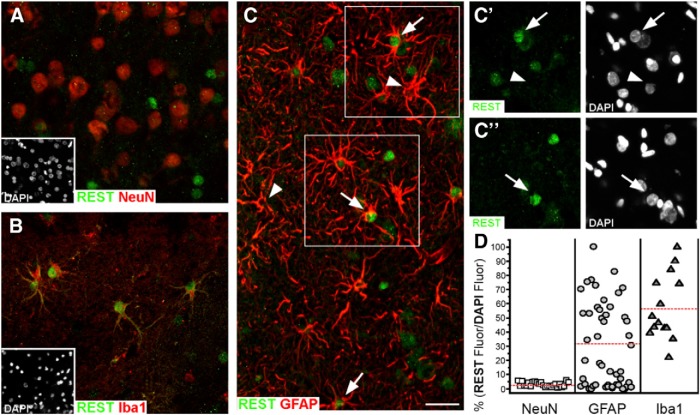
Expression of REST in various cell types in the human brain. Sequential slices were dually immunolabeled with an anti-REST antibody (green) together with antibodies against markers of various cell types (red). The REST immunolabeling is different in the various panels. ***A***, In neurons, no appreciable REST immunolabeling is present. ***B***, In microglia, nuclear REST immunolabeling is strong. ***C***, In astrocytes, nuclear REST immunolabeling is variable. ***C'***, ***C''***, Enlargements of the immunolabeling in the boxes in ***C***, confirming the variable REST immunolabeling in the nuclei of astrocytes. ***D***, Quantification of the nuclear REST data illustrated in ***A–C*** is shown. Scale bar: (in ***C***) ***A–C***, 30 μm. The figure is from Prada et al., 2011.

The downregulation of REST is critical for the acquisition and preservation of neuronal specificities. The expression of REST-dependent genes activates a variety of important processes during advanced differentiation, such as axonal growth, the establishment of synaptic contacts, and membrane excitability (Paquette et al., 2000; Aoki et al., 2012). In the adult brain, all neurons exhibit low levels of REST (Fig. 1), although with some moderate heterogeneity (Palm et al., 1998; Calderone et al., 2003; Sun et al., 2005; Gao et al., 2011). Compared to the case in stem cells, the number of REST target genes is lower in adult neurons. The decrease of these genes, which is established during differentiation, is mostly a consequence of chromatin remodeling that reduces gene access to transcription (Murai et al., 2005; Abrajano et al., 2009; Yoo et al., 2009; Juliandi et al., 2010, Johnson et al., 2012).

Despite the low level of REST that is typical of adult neurons, the average level in whole-brain tissue is considerable. In fact, REST levels are high in most non-neuronal glial cells (Prada et al., 2011; Fig. 1), in endothelia and other cells of vessels, and in neural stem cells concentrated in specific areas (dentate gyrus of the hippocampus, subventricular zones, and a few others) where neurogenesis takes place (Gao et al., 2011). Outside the brain, low REST levels are typical of many neural cells. Other such cells, for example, many neuroblastoma cells, exhibit high REST levels; however, these levels decrease to neuronal levels upon long-term treatment with retinoic acid (Singh et al., 2011). In contrast, the levels of REST remain high in non-neural cells during and after differentiation. Downregulation in cells of this type occurs only in a fraction of tumors, where REST contributes to increased proliferation (Westbrook et al., 2005; Wagoner et al., 2010; Negrini et al., 2013a).

## Physiology

The hypothesis that low levels of REST are necessary in neurons to enable the transcription of neural genes, which was put forth by Chong et al. (1995) in their discovery article, was supported and strengthened by many subsequent studies. By fine-tuning gene expression, REST also participates in chromatin plasticity (Ballas et al., 2005; Rodenas-Ruano et al., 2012). Many genes expressed in adult neurons are governed or related to REST. These include genes for transcription factors, dependent on REST for their repression, such as *Sp1*, *Grin1*, *Ascl1*, *Isl1*, and many others, as well as genes that are not repressed. The functions of these genes are influenced by REST, and this type of gene includes *Creb*, *E47*, *Poh3f2*, *Gata2*, *Myod*, and many others (Wu and Xie, 2006; Johnson et al., 2008; Moreno-González et al., 2008; Yuan et al., 2013; Bersten et al., 2014).

Among the other genes largely repressed by REST are those encoding for many channels and transporters, as well as presynaptic and postsynaptic proteins. These encoded proteins, in turn, ensure membrane excitability and synaptic transmission. The REST dependence of voltage-gated Na^+^ channels, which was already reported at the time that REST was discovered (Chong et al., 1995), has been confirmed several times (Nadeau and Lester, 2002; Drews et al., 2007; Pozzi et al., 2013). Additional channels, specifically Ca^2+^ and K^+^ (Ariano et al., 2010; Ono and Iijima, 2010; Uchida et al., 2010; van Loo et al., 2012) and the hyperpolarization-activated, cyclic nucleotide-gated channel HCN1 (McClelland et al., 2011a), are all REST dependent. Likewise, the upregulation of the chloride transporter KCC_2_ in adult cortical neurons relies on low REST levels. This transporter is critical for the Cl^−^ switch that converts the function of GABA from excitatory to inhibitory (Uvarov et al., 2005; Yeo et al., 2009). In addition, REST governs the expression of critical subunits of nicotinic and glutamatergic (NMDA and AMPA) receptors (Qiang et al., 2005; Moreno-González et al., 2008; Rodenas-Ruano et al., 2012), as well as of a few G-protein-coupled receptors (Kim et al., 2004; Formisano et al., 2007; Henriksson et al., 2014). Particularly interesting are the neurotrophins and their receptors. The repression of BDNF, which is induced by REST increases, has been envisaged as a potential risk for neuronal cells (Zuccato et al., 2007; Hara et al., 2009; however, see Garriga-Canut et al., 2006). Among the Trk receptors, the REST dependence of TrkC is also relevant for cancer development (Mulligan et al., 2008; Lawn et al., 2015). The common neurotrophin receptor, p75^NTR^, is also repressed by REST (Nakatani et al., 2005; Negrini et al., 2013b).

One of the neuron-specific processes controlled by REST is transmitter release. In this case, REST represses the genes that encode for synaptic vesicle proteins and those involved in vesicle exocytosis. These proteins include the specific SNAREs, neurotransmitter transporters, and other membrane proteins, and the proteins accumulated in the lumen (D’Alessandro et al., 2009) or exposed to the cytosolic surface of vesicles, such as the most abundant protein, synapsin 1, which plays a crucial role in vesicle traffic and recycling (Schoch et al., 1996; Paonessa et al., 2013).

Other processes are important for REST physiology. Trafficking of the factor to the nucleus is needed for it to function. The process that has been specifically investigated (Shimojo, 2006) is the accumulation of the repressor in the nucleus, the compartment of its action. However, in various diseases such as Alzheimer’s disease and Huntington’s disease, the transport of REST to the nucleus is altered in various ways (for stimulation, see Zuccato et al., 2007; for depression, see Lu et al., 2014). A specific, important aspect of REST downregulation in adult neurons is its effects on miRNA expression, in particular miR-124, miR-9/9*, and miR-132, the expression of which is stimulated by another transcription factor, CREB (Wu and Xie, 2006), and is indeed reciprocal to REST. Therefore, the three miRNAs, which are abundant in resting neurons, decrease upon stimulation, causing REST levels to increase (Conaco et al., 2006; Wu and Xie, 2006; Sanuki et al., 2011; Sun et al., 2013). Each one of the miRNAs mentioned above has several functions, including, for miR-132, the regulation of neural tissue plasticity, synaptogenesis, and synaptic function (Siegel et al., 2011); and for miR-124 and miR-9/9*, the promotion of neuronal migration (Volvert et al., 2014) and the assembly of the ATP-dependent, chromatin remodeling BAF (Brahma-associated factor) complexes, which are essential for learning and memory (Yoo et al., 2009; Sun et al., 2013; Vogel-Ciernia et al., 2013). REST dependence is not restricted to miRNAs but extends to other types of neurally restricted, ncRNAs (Rossbach, 2011). A small dsRNA acts by competing with REST for target gene function (Kuwabara et al., 2005; Yoo et al., 2009). Moreover, a fraction of the long noncoding ncRNAs, which have neurally restricted expression, is also transcriptionally controlled by REST. Whether such REST control is relevant for neuronal cell physiology is unclear because the general function of ncRNAs also remains to be established (Johnson et al., 2009, 2014).

REST levels also control factors involved in alternative mRNA splicing, contributing significantly to neuronal specificity. For instance, REST controls the expression of nSR100, which operates on a large panel of mRNAs that are differentially spliced in neurons and non-neural cells (Raj et al., 2011). The mRNA for REST is among these. In neurons, REST frequently appears in its truncated, inactive form, REST4, which competes with full-length REST for the binding of target genes. Of note, the coexistence of the full length and the truncated forms attenuates the repression of REST target genes, and thus protects neuronal cells (Raj et al., 2011). Protection by REST4 was confirmed by studies of ethanol intoxication in control and REST knock-out mice (Cai et al., 2011). nSR100 also operates together with other neuron-specific, REST-dependent splicing factors. Its combination with Nova1 governs the splicing of the demethylase LSD1, a component of REST operative complexes. Short LSD1, the predominant form in neurons that results from mRNA splicing by the two factors, favors the disassembly of REST complexes. The final result of this dual splicing therefore mimics the effect of REST4 (Rusconi et al., 2014). Another REST-dependent factor, Nova2, induces the splicing of a key neural adhesion protein, L1CAM. While neurons express the full-length form of L1CAM, many non-neural cells express the shorter form, which exhibits poor homotypic binding and transmembrane signaling capacity (Mikulak et al., 2012). Because of its ability to control the expression of splicing factors, the number of REST-dependent gene products is much larger than the number of its direct gene targets. Accordingly, repression of the *nSR100 and NOVO* genes by increases in REST does ultimately alter the structure and function of whole synapses (Ule et al., 2005; Eom et al., 2013).

The level of REST in adult neurons is not always low. REST levels have been reported to increase progressively in the nuclei of hippocampal and brain cortical neurons in healthy, aging humans, possibly because of the increased frequency of stress and Wnt-controlled signaling. These findings correlate well with the upregulation of various protective genes and the downregulation of potentially toxic genes, resulting in the preservation of global cognition and increased neuronal longevity (Lu et al., 2014). Of note, nuclear REST levels do not increase in aging humans with brain diseases, especially in patients with Alzheimer’s disease (see the discussion in the Alzheimer’s disease section).

**Figure 2:**
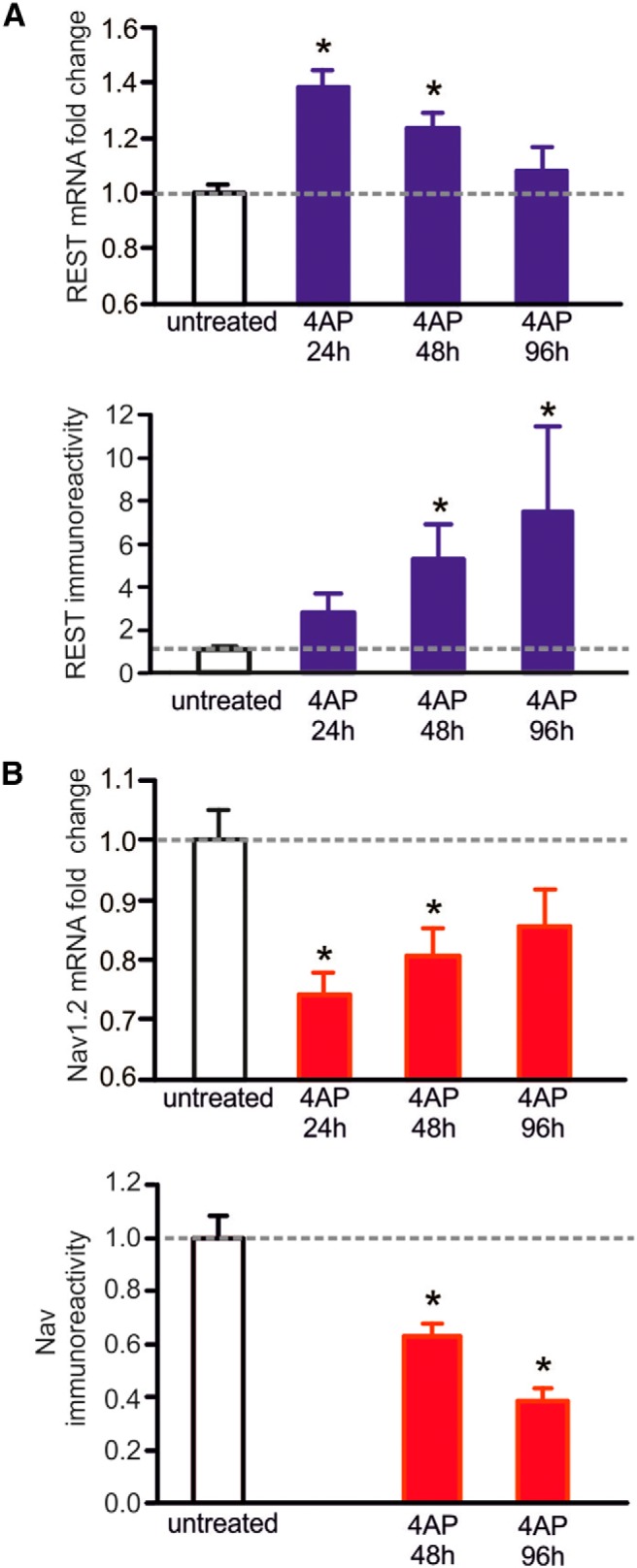
4-Aminopyridine (4AP)-induced cortical neuron hyperactivity increases REST expression and, in parallel, downregulates the expression of the Na^+^ channel Na_v_1.2. ***A***, Analysis of REST (blue). Top, Quantitative RT-PCR analysis of REST mRNA levels in cortical neurons that were either untreated or treated with 4AP (100 μm) for 24, 48, and 96 h. Bottom, Changes in the REST protein of cortical neurons treated as in the top panel, quantified by Western blotting. ***B***, Analysis of Na_v_1.2 (red). Top, Quantitative RT-PCR of the changes in Na_v_1.2 mRNA in cortical neurons, which were untreated or treated with 4AP as in ***A***. Bottom, Na_v_1.2 protein of cortical neurons treated as in ***A***, quantified by Western blotting. Notice that, for both mRNA and protein, the opposite changes were induced by 4AP: an increase in REST mRNA at 24 h, followed by a decrease back to the untreated level at 96 h, accompanied by a decrease of Na_v_1.2 mRNA, and followed by an increase at the same times; and a slow increase in REST protein (up to approximately eightfold at 96 h) accompanied by a slow decrease in Na_v_1.2 (∼40% at 96 h). The data in the columns in ***A*** and ***B*** (mean ± SEM) were obtained from seven to eight (top) and four (bottom) samples from two separate neuronal preparations. *p < 0.05, Kruskal–Wallis test followed by Dunn's test versus untreated. The figure is from Pozzi et al., 2013.

Neuronal excitation is also controlled by REST. Prolonged *in vitro* depolarization of neuronal primary cultures with high extracellular K^+^ was reported to induce increases in REST accompanied by the downregulation of proteins encoded by target genes, including the neurotrophin BDNF (Hara et al, 2009) and the transcription factor NPAS4 (Bersten et al., 2014). Similar results were obtained when depolarization was induced in primary cultures of mouse hippocampal neurons by up to 4 days of treatment with 4-aminopyridine, a blocker of K^+^ channels. The treatment, the effects of which were analyzed by patch-clamp and multielectrode array recordings, was shown to induce, in excitatory neurons, a transient increase in REST mRNA followed by an increase in the protein (Fig. 2*A*) and a progressive decline in action potential frequency (Pozzi et al., 2013). The effect was due to the decrease in both the firing frequency and density of Na^+^ channels, which were identified as Na_v_1.2 channels (Fig. 2*B*). This decreased excitability corresponds to the decline of a well known neuronal condition, intrinsic homeostasis (Pozzi et al., 2013).

Studies of G-protein-coupled receptors have not included details about REST increases and gene target inhibition (Kim et al., 2004; Formisano et al., 2007; Henriksson et al., 2014). In contrast, studies with kainate, a glutamatergic agent that is active on channel receptors, that were first performed by Palm et al. (1998) and then by other groups (see, among others, Calderone et al., 2003; Spencer et al., 2006; Formisano et al., 2007; McClelland et al., 2011a, 2014; Orta-Salazar et al., 2014; Rivera-Cervantes et al., 2015), revealed the induction of REST upregulation *in vivo* in hippocampal and cortical neurons. Analogous results induced by prolonged treatment with the same agent were obtained in primary cultures of rat hippocampal CA1 neurons (Spencer et al., 2006; Lau and Tymianski, 2010; Ortuño-Sahagún et al., 2014; Rivera-Cervantes et al, 2015) and in brain slices prepared *ex vivo* (Calderone et al., 2003; McClelland et al., 2011a).

**Figure 3:**
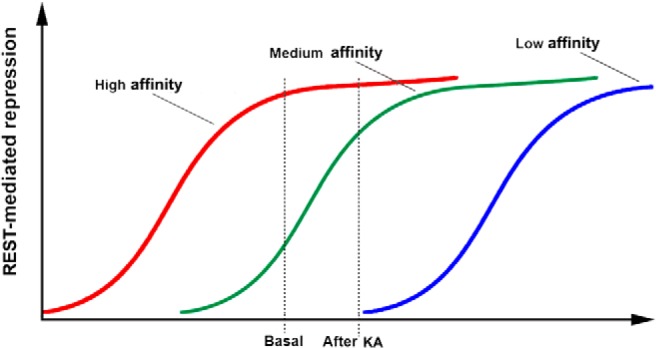
REST binding affinity dependence of neuronal gene repression. The figure illustrates the average changes in the repression induced by increases in REST levels in three groups of RE-1-positive genes, which were dependent on their average binding affinity. The genes with high affinity (red) are already 100% repressed at basal levels of REST. The genes with intermediate affinity (green), which are ∼30% repressed at basal levels, exhibit an increase in their repression to ∼80% upon cell treatment with kainate (KA; 6 μm). The low-affinity genes are repressed neither at basal levels nor after KA and would require higher levels of REST to be repressed. The figure is from McClelland et al., 2014.

The majority of the studies reported to date have focused on specific processes of REST increases. They demonstrated the relevance of REST and provided information about the mechanisms involved. In these studies, however, comprehensive analysis of the changes in gene expression induced by the increased levels of REST was not performed. Detailed information about this issue was first provided by a recent study performed by McClelland et al. (2014), in which mice were exposed to kainate. In this study, the expression of >400 classic REST gene targets, including the RE-1 sequence in their regulatory domains, was investigated after increases in REST levels of a few fold. The expression of only a relatively small (∼10%) fraction of these genes, characterized by intermediate affinity for the repressor, was found to significantly decrease (McClelland et al., 2014). Analysis of the genes of this fraction revealed that they encode voltage-gated and receptor channels (including, among others, Na^+^ and K^+^ channels, glutamatergic receptor subunits, the HCN1 voltage-gated channel, and the Cl^−^ transporter KCC2), together with signaling proteins, some kinases, transcription factors, and a few other proteins (McClelland et al., 2014). The genes with high and low binding affinity, which were already demonstrated to exhibit considerable and minor repression, respectively, before stimulation, were found to undergo no appreciable change after kainate stimulation (Fig. 3). The REST dependence of the results obtained with the various target genes was demonstrated by experiments in which the tone of the repressor was attenuated by the introduction of decoy oligodeoxynucleotides comprised of the RE-1 binding sequence (McClelland et al., 2014). Currently, not all of the genes that modify their expression following stimulation-induced changes in REST may have been identified. Yet, the results of the McClelland et al. (2014) study are expected to have important implications for the interpretation of the REST target gene expression results. It is clear, in fact, that the genes affected by stimulation-induced increases in REST are numerous and that many of them are functionally relevant. At least some of the proteins encoded by these genes could operate not separately but coordinately with each other.

A final problem to be considered addresses the possible role of REST in excitotoxicity, a process of neuronal death that is known to be induced by glutamate and its analogs (Spencer et al., 2006; Lau and Tymianski, 2010; Pozzi et al., 2013; Ortuño-Sahagún et al., 2014; Rivera-Cervantes et al., 2015). Two findings may be important when discussing the possible contribution of REST to this process. First, the excitotoxicity elicited by glutamatergic agents reported in the studies described above was not reported in others (Palm et al., 1998; McClelland et al., 2011a). This variability has been proposed to depend on a protective intracellular MAPK–ERK signaling cascade that is triggered by the same glutamatergic agents (Ortuño-Sahagún et al., 2014). Second, depolarizing agents such as high K^+^ and 4-aminopyridine, which induce increases of REST analogous to those of glutamatergic agents, do not induce any excitotoxicity (Hara et al, 2009; Pozzi et al. 2013; Bersten et al., 2014). Based on these considerations, the role of REST appears to consist not in the induction but at most in the strengthening of the excitotoxic effects induced by the glutamatergic agents.

## Pathology

In the last paragraph of the previous section, we concluded that REST may not play a dominant role in the excitotoxicity triggered in neurons by glutamatergic agents. Nevertheless, the possibility of a contribution of REST to the toxicity induced by glutamate (Spencer et al., 2006; Pozzi et al., 2013) or polychlorinated biphenyls (Formisano et al., 2014) appears quite likely. A similar conclusion may also be valid for a neural cell line exposed to oxygen–glucose deprivation. In the cells of this line, an increase in REST was shown to act via antagonism of the well known transcription factor CREB (Wu and Xie, 2006), with ensuing downregulation of CART (cocaine and amphetamine-regulated transcript), a secretory peptide known to exert neuroprotective effects (Zhang et al., 2012).

The most detailed studies emphasizing the role of REST in brain pathology were performed in rats exposed to global brain ischemia due to *in vivo* electrocauterization of vertebrate arteries followed by occlusion of the carotid arteries (Calderone et al., 2003). These treatments generate a condition similar to that of stroke. In light of this similarity, the data from these studies will be attributed to this type of pathology. In the first stage, successive increases in REST mRNA and protein were found to occur in CA1 pyramidal neurons of organotypic hippocampal slice cultures 24-48 h after stroke. The increase in REST was shown to downregulate the expression of various targets, including the gene encoding for the GluR2 subunit of the glutamatergic AMPA receptor (Calderone et al., 2003; Jia et al., 2006). Death of the insulted CA1 neurons was found to take place 7 d after stroke. Neither the increase in REST nor the subsequent death observed in CA1 occurred in other hippocampal neurons, such as CA3 pyramidal neurons and the granule neurons of the dentate gyrus (Calderone et al., 2003).

Further studies performed by the same research group investigated the mechanisms of the CA1 neuronal death associated with the increase in REST induced by stroke. In addition to the AMPA subunit, stroke was shown to affect the NMDA receptor by inducing the replacement of the GluN2B subunit with the GluN2A subunit. These changes were accompanied by a selective increase in synaptic transmission (Rodenas-Ruano et al., 2012). The REST increase was found to occur only in animals that did not express casein kinase 1, an enzyme that participates in the regulation of the ubiquitin-based degradation of REST (Kaneko et al., 2014). The number of genes downregulated by the increase of REST in these neurons was relevant (Noh et al., 2012) and included the μ *opioid receptor* gene (Formisano et al., 2007). Among these genes was one encoding for miR-132, which appeared to be causally related to neuronal death (Hwang et al., 2014). Unexpectedly, the repression of miR-132 was not accompanied by changes in miR-124 and miR-9/9* (Hwang et al., 2014), two neuronal miRNAs that are also expected to operate reciprocally to REST (Conaco et al., 2006). Together, the data presented in this section suggest that the increases in REST induced by stroke or toxic treatments contribute to neuronal death by affecting the expression of numerous genes and their encoded proteins; whereas, other, non-death-inducing genes may be changed less or not at all.

## Diseases

### Brain diseases

In light of the key role of REST in brain development and function, it is not surprising that it also plays mechanistic roles in the pathogenesis of several brain diseases. REST is also known to be involved in the biogenesis of brain tumors; however, this will not be discussed here as it was recently reviewed elsewhere (Huang and Bao, 2012; Negrini et al., 2013a). Rather, the following sections will summarize the present knowledge of the role of REST in several brain diseases as well as in terms of therapy.

### Psychiatric diseases

#### Schizophrenia

The pathogenesis of schizophrenia, the most important psychiatric disease, is widely believed to include the deregulation of a number of genes, the so-called schizophrenia-associated genes, which have been identified by genome-wide approaches. One of these associated genes encodes for SMARCA2, a member of the switch/sucrose nonfermentable (SWI/SNF) chromatin-remodeling complex that is known to operate under the control of REST. Studies in neural cell lines and a transgenic mouse model showed that the interaction between SWI/SNF and REST can attract 80% of the other associated genes, suggesting the possible role of a large, REST-containing complex in the so-called genetic architecture of schizophrenia (Loe-Mie et al., 2010).

#### Other mental diseases

Several diseases of this group are based on classic genetic mechanisms. X-linked mental retardation was reported to depend on SMCX demethylase 5C, an enzyme that demethylates the K4 site of histone H3, which is critical for the activity of the REST complexes. Loss of the SMCX enzyme was found to impair REST-mediated downregulation of many neuronal genes, thus contributing significantly to the mechanism of the disease (Tahiliani et al., 2007). Another frequent form of mental retardation is part of Down syndrome, which results from trisomy of chromosome 21. The alteration of REST function reported in this disease was found to depend on overexpression of the protein DYRK1A, which is encoded by a gene of the trisomic chromosome. Work in various transgenic mouse models showed that DYRK1A binds SWI/SNF, the chromatin-remodeling complex regulated by REST, and is also involved in the pathogenesis of schizophrenia. In adult mouse neurons, the DYRK1A binding protein was shown to cause an increase in REST, with ensuing alterations in gene expression, and a severe reduction in dendritic growth and complexity. When the *Dyrk1a* gene was downregulated, these alterations were prevented. The conclusion is that REST, via its regulation of the SWI/SNF chromatin complex, contributes significantly to the neural phenotypic changes that characterize the disease (Canzonetta et al., 2008; Lepagnol-Bestel et al., 2009).

Dynorphin is an endogenous, proteolytically processed opioid protein known to give rise to many secretory peptides (β-neoendorphin, dynorphin, leu-enkephalin, and a few others) that are all ligands of the κ-type opioid receptor. Expression of these peptides has been found to be altered in the brains of many patients with mental disorders, including drug addiction and some forms of schizophrenia (Tejeda et al., 2012). REST is known to repress the expression of most secretory proteins (D’Alessandro et al., 2009). The hypothesis of this disease is that mental disorders are elicited by the decreased expression of opioid proteins and peptides induced by the upregulation of REST, possibly as a consequence of the decreased expression of the reciprocal miRNAs miR-132, miR-9/9*, and/or miR-124 (Henriksson et al., 2014).

### Neurological diseases

#### Alzheimer’s disease

For many years, intensive studies of the mechanisms of the pathogenesis of Alzheimer’s disease, which affects a considerable fraction of elderly humans, did not consider the possible involvement of REST. Interestingly, however, various factors now recognized as being REST dependent, such as miRNAs (including miR-9/9* and miR-132, which operate reciprocally to REST) and ncRNAs, have been proposed to play significant roles in Alzheimer’s disease (Schonrock et al., 2012; Lau et al., 2013; Wu et al., 2013).

Two interesting studies recently rejuvenated interest in the role of REST in Alzheimer’s disease. In the first study, investigation of brains from patients and a specific transgenic mouse model revealed considerable increases in REST with concomitant decreases of choline acetyltransferase (the key enzyme of acetylcholine biosynthesis) in neurons in the area of the nucleus of Meynert and in fibers traveling to the frontal and motor cortices, and to hippocampal area CA1. In light of the REST dependence of choline acetyltransferase expression and the relevance of cholinergic neurons in the development of the disease, the increase in REST was hypothesized as a mechanism of the induction of the overexpression and accumulation of β-amyloid and other proteins involved in neuronal degeneration (González-Castañeda et al., 2013; Orta-Salazar et al., 2014).

The other study, performed in humans and mouse models, demonstrated that in Alzheimer’s disease and other neurodegenerative diseases, such as frontotemporal dementia and dementia with Lewy bodies, the level of REST in neurons of the hippocampus and frontotemporal cortices does not increase in the nuclei, as observed in healthy aging humans, but remains in the cytoplasm, where it accumulates within autophagosomes together with misfolded proteins (Lu et al, 2014). In other areas of the brain, such as the dentate gyrus and the cerebellum, that are not affected by Alzheimer’s disease, the concentration of REST in nuclei was as high as in control subjects. Together, the results obtained by Lu et al. (2014) in the brains of healthy humans and Alzheimer’s disease patients suggest that REST plays a critical role in neuronal viability. The development of neurodegenerative diseases may thus be favored by the expression of genes and processes related to neuronal degeneration, which, in the brains of healthy, aging humans, may be repressed by REST (Lu et al., 2014).

#### Parkinson’s disease

Compared to the studies on Alzheimer’s disease, those related to the role of REST in the Parkinson’s disease appear less conclusive. REST was first investigated by treating a human dopaminergic neural cell line, SH-SY5Y, with MPP^+^, a neurotoxin known to affect dopaminergic neurons. In these studies, REST was found to elicit deleterious effects on SH-SY5Y cell viability (Yu et al., 2009). Subsequent analysis in a spontaneous autosomal-recessive rat model revealed the deregulation of several miRNAs, in particular of miR-132, followed by the degradation of dopaminergic neurons in the midbrain. In light of its reciprocal operation with miR-132, levels of REST were probably increased, as apparently confirmed by the accompanying decrease of BDNF, a neurotrophin encoded by a REST target gene. These results were interpreted as a suggestion of the involvement of a decrease in miR-132, and possibly an increase in REST, in the degradation of Parkinson’s disease-specific neurons (Siegel et al., 2011; Lungu et al., 2013).

On the other hand, in recent experiments performed in REST knock-out mice, the administration of MPTP, a toxin known to elicit the appearance, in both humans and animals, of parkinsonian disorders due to the degeneration of dopaminergic neurons in the striatum, induced dramatic losses of those neurons. These results were interpreted as depending on the high vulnerability of the striatal neurons in knock-out mice (Yu et al., 2013). However, as discussed in detail in the Epilepsy section, the results induced in the neurons of REST knock-out animals are complex, involving the cooperation of numerous target genes. Therefore, the interpretation of the data by Yu et al. (2013) remains unclear.

#### Huntington’s disease

In this disease, the neurodegeneration, which affects the basal ganglia and cerebral cortex in particular, is widely attributed to the mutant form of a specific protein, huntingtin. The mutant form is characterized by the extension of the N-terminal polyglutamine repeat. After investigations in animal models and in human brains postmortem, the disease was initially proposed to be a consequence of the accumulation of REST in the nuclei of neurons, which was favored by the huntingtin mutant and resulted in the repression of important target genes, such as the one encoding the neurotrophin BDNF (Zuccato et al., 2003). The attenuation of REST expression in neurons was found to restore BDNF levels, confirming the REST dependence of the aberrant gene transcription in this disease (Zuccato et al., 2007). In addition, mutant huntingtin has been found to induce effects other than the decreased expression of BDNF. In particular, Sp1, a transcription factor that has a role in the control of *Rest* gene expression, was found to be upregulated (Ravache et al., 2010). Concomitant investigation of thousands of direct target genes of REST identified dysregulation, not only in mRNAs and proteins but also in other RNAs, including both miRNAs and long ncRNAs (Bithell et al., 2009; Buckley et al., 2010). Abnormal changes, not only transcriptional but also epigenetic, have also been emphasized in the brains of Huntington’s disease patients (Bithell et al., 2009; Buckley et al., 2010). Finally, recent results have revealed a variety of additional defects, including increased autophagy (Martin et al., 2015), altered mTORC (mammalian target of rapamycin complex 1) metabolism (Lee et al., 2015), and enhanced microglial reactivity (Crotti et al., 2014) in the neurons of Huntington’s disease patients. Whether REST has a role in these altered processes remains to be investigated.

#### Epilepsy

Epilepsy, the third most common disease of the human brain, is the one in which the role of REST has been investigated in the most detail. Epilepsy is known to develop when the brain is injured or when the tendency to generate seizures evolves into an enduring process that is inextricably distributed among neuronal circuits, converging at the level of dysfunction. This conversion appears to be sustained by epigenetic alteration of important genes (Jessberger et al., 2007; McClelland et al., 2011a,b; Roopra et al., 2012; Goldberg and Coulter, 2013). REST and changes in the expression of its target genes have been envisaged as critical events governing later development of the disease. However, the mechanisms involved are still being debated.

In the literature of epilepsy, a few reports have emphasized the role of REST in the induction of the disease and possibly also in protection from it. Ten years ago, a 2-deoxy-d-glucose ketogenic diet was reported to have an antiepileptic effect via the activation of a chromatin remodeling complex controlled by an increase in REST (Garriga-Canut et al., 2006). Recently, however, the ketogenic diet has been shown to maintain its antiepileptic effect even in the absence of the REST increase (Hu et al., 2011b).

Additional studies found that the susceptibility to kindling and enhanced mossy fiber sprouting was greatly worsened in mice bearing a conditional deletion of REST in forebrain neurons. Based on these results, the REST increases induced by seizures were suggested to operate favorably in the patients, slowing down the development of epileptogenesis in the limbic cortex (Hu et al., 2011a). Further experiments in conditional knock-out mice were performed on neurons of the forebrain. Using the pentylenetetrazole model of acute seizures, the authors observed that in the knock-out mice, higher doses of the drug were needed to induce tonic convulsions and death (Liu et al., 2012). Although these studies were technically convincing, their interpretation remained open to question. In fact, the deletion of REST in neurons that have not started epileptogenesis is expected to induce the expression of large numbers of target genes, including those bound by REST with high affinity (Fig. 3), which in control neurons remain at least partially repressed even at rest (McClelland et al., 2014). Therefore, the increased epileptic responses observed in REST conditional knock-out mice could be due to genes not involved in the regular epileptogenic process.

On the other hand, McClelland et al. (2011a) reported exciting results regarding the role of HCN1, a hyperpolarization-activated, cyclic nucleotide-gated channel. HCN1 is known to play major roles in the control of neuronal excitability, synaptic transmission, and oscillatory activity, in both single neurons and neuronal networks. In the mouse model of temporal lobe epilepsy induced by kainate, the expression of HCN1 and the activation of specific currents were repressed by an increase in REST (McClelland et al., 2011a). Subsequent studies revealed that the repression resulting from an increase in REST was not limited to HCN1 but also included 10% of the analyzed target genes, including those encoding other channels and signaling proteins (see details in the Physiology section). The attenuation of REST binding to the RE-1 sequence of the DNA of its target genes via specific decoy oligonucleotides was found to elicit not only a restoration of specific currents but also a reduction of the initial pattern of seizures (McClelland et al., 2014).

**Figure 4:**
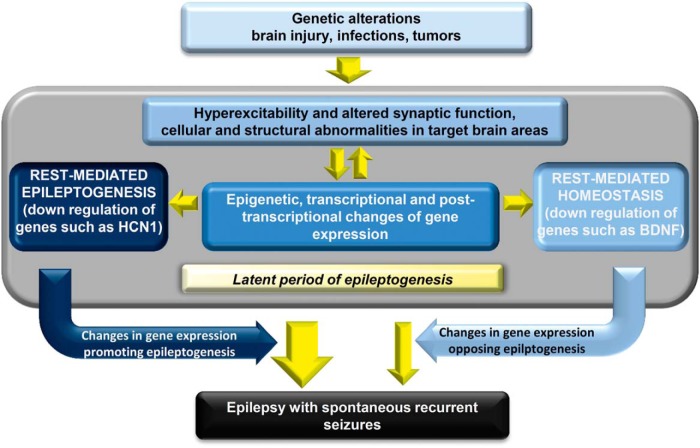
Epileptogenesis: sequence of events, including the role of REST. Causal processes that are possibly involved in the beginning of epileptogenesis are listed in the azure box at the top. Several boxes included in the middle profile (gray) summarize the subsequent events. Hyperexcitability and other structural and functional alterations (top, azure box) are directly connected to the causal processes at the top and to the changes in gene structure/expression of the central box. The appearance of epilepsy, however, is not rapid. Time is needed to convert the first seizures to an enduring process distributed to neuronal circuits. This maturation is not described; instead, it is just mentioned in the thin yellow box at the bottom of the green profile. The gene expression events governed by REST are shown in the two boxes connected to the central box. The blue box to the left includes the predominant genes whose repression promotes epileptogenesis; the clear azure box to the right includes the genes that tend to maintain cell homeostasis and thus to negatively modulate epileptogenesis. The difference in the relevance of the two modulations of REST-dependent genes is illustrated by the different thicknesses of the yellow arrows that receive their modulation. The resulting ongoing epilepsy is indicated in the black box at the bottom.

In conclusion, the results of studies of the role of REST in epilepsy might appear contradictory. It should be emphasized, however, that conditional deletion of REST before epileptogenesis induces the overexpression of many genes (Hu et al., 2011a; Liu et al., 2012), which may differ in various cell types. In contrast, the protective role of the attenuation of REST activity, as shown by McClelland et al. (2014) after the induction of status epilepticus, clearly documents the participation of increased REST levels in the establishment of the enduring process. This role of REST most likely includes epigenetic effects on many genes with intermediate affinity for the repressor. The overall range of events occurring during epileptogenesis, including the possibly variable role of REST in this process, is illustrated in Figure 4.

## Therapy with drugs related to REST

In light of the relevance of REST in the physiology and pathology of adult neurons, it is not surprising that investments have been made toward the development of specific drugs that could function in a few of the diseases presented so far. At the moment, however, only two approaches have been developed toward translation. The first involves the use of analogs of valproic acid, a drug identified a few decades ago that is used in therapy for various forms of epilepsy. The actions of valproic acid were initially attributed to an enhancement of central GABAergic neurotransmission, possibly combined with the inhibition of Na^+^ channels (Tunnicliff, 1999). It should be noted, however, that valproic acid, which is a high-affinity inhibitor of histone deacetylases, is not specific for the forms 1 and 2 of the enzyme, which are critical for the transcriptional repression by REST. It also acts on the other 16 forms of the enzyme, which are involved in many additional functions (Göttlicher et al., 2001). Studies were therefore started, and are still being performed, to identify new drugs that work with mechanisms analogous to those of valproic acid but that are specific to deacetylases of the first class. Because of their specificity, these drugs, and possibly also their inhibitors, might be used in therapies for numerous brain defects and diseases, including neurodegenerative disorders as well as defects of learning and memory, and of cognition (Gräff and Tsai, 2013; Penney and Tsai, 2014; Didonna and Opal, 2015).

A second approach was developed for the treatment of Huntington’s disease. As specified in a preceding section, one of the mechanisms contributing to Huntington’s pathogenesis is the increased accumulation of REST in neuronal nuclei, possibly due to a peculiar function of the mutant huntingtin protein that reinforces the transport of the repressor across the nuclear envelope (Zuccato et al., 2003, 2007). The results of experiments in Huntington’s disease cell cultures transfected with a dominant-negative REST construct had already documented the importance of the elevated nuclear REST levels and the resulting increased repression of target genes in the diagnosis of the disease (Zuccato et al., 2007). Further evidence along this line has been generated more recently by the use of decoys, double-stranded oligodeoxynucleotides that compete with REST for binding to the RE-1 sequence of DNA (Soldati et al., 2011). Decoys were also found to attenuate the repression of gene expression induced in neurons by kainate treatment (McClelland et al., 2014). In the meantime, two pharmacological approaches have been developed based on the selection, by high-throughput screening, of drugs that were later analyzed in various cell lines and *in vivo* in animals. The first drug was found to reduce nuclear accumulation of mSin3b, a corepressor of the REST N-terminal operative complex (Conforti et al., 2013), and the second drug was found to decrease cell levels of REST by stimulating its degradation (Charbord et al., 2013). Upon application of either one of these drugs, various REST target genes were shown to increase their levels. In the future, other drugs that interfere with the role of REST in neurons are expected to be developed. The interest in such developments could involve not only Huntington’s disease but also other brain diseases in which changes in REST are critical for pathogenesis.

## Concluding remarks

During the last 15 years, growing interest in the role of REST in adult neurons has paved the way to the identification of multiple, highly interesting processes that take place in the physiology and pathology of the brain as well as in several brain diseases. Although the number of REST-repressed genes is lower in adult neurons than in neural stem cells and early precursors (Sun et al., 2005; Greenway et al., 2007; Johnson et al., 2008), the functional relevance of most of them is considerable. Therefore, the identification of REST as a master factor, which was initially proposed for differentiating precursors (Ballas and Mandel, 2005), appears to be appropriate for adult neurons as well.

Among transcription factors, REST exhibits several unique properties. The very low levels of REST, which are initially established during differentiation, are maintained in adult neurons by controlled transcription of the *Rest* gene, coupled to the very active ubiquitination and ensuing proteolysis of the REST protein. The unusual length and repetitive structure of RE-1, the DNA sequence of REST binding in many target genes, ensures that the repressor has highly specific actions. Additional binding sequences are now under investigation; therefore, a report regarding their properties would be premature. The unique properties of REST and its actions, which are important in adult neurons, appear destined to undergo further development in the near future. Among the examples of important properties being investigated now and in the near future, a few are presented here.

High expression of many REST-dependent genes in neurons was shown to be reinforced by splicing mechanisms, such as generation of the inactive, truncated form of REST, REST4 (Raj et al., 2011). Strengthening of low REST action by REST4 has been confirmed by the study of ethanol intoxication (Cai et al., 2011). In the future, this reinforcement could be further increased. In fact, additional REST-dependent splicing factors are expected to be identified. The same is true for ncRNAs. At the moment, we are aware of three miRNAs that operate reciprocally to REST (Conaco et al., 2006). Whether other miRNAs and ncRNAs participate in the regulation of repressor function in neurons is still unknown (Johnson et al., 2009, 2014).

With respect to gene transcription, REST is highly efficient. Therefore, the repression of single target genes is usually thought to be its only function. Among other transcription factors, however, cooperation in the expression of single genes is quite common. In the case of REST, a cooperation that is widely accepted is with the Polycomb complexes (Arnold et al., 2013; Dietrich et al., 2012); however, additional instances of cooperation appear to exist (Wu and Xie, 2006; Johnson et al., 2008) and might be identified soon. REST-mediated repression of target genes encoding transcription factors is also highly interesting. When the transcription factors involved are other repressors, REST is expected to counteract the repression of their targets; if the transcription factors are stimulatory, REST is expected to indirectly extend the repression of their target genes. Currently, only a few transcription factors known to play a role in the indirect actions of REST have been identified (Ooi and Wood, 2007; Jobe et al., 2012). New factors are therefore expected to be identified soon.

Highly interesting has been the identification of intermediate affinity REST targets, such as the genes that are downregulated upon small increases in the repressor (McClelland et al., 2014). These genes, which comprise ∼10% of the investigated targets expressing RE-1 sequences in their promoter or other regulatory areas, may be only a fraction of the number of genes that are downregulated by an increase in REST. In fact, other REST target genes are known to express binding sequences different from RE-1 (ENCODE Project Consortium, 2012). Moreover, additional REST target genes could be repressed/stimulated by indirect mechanisms (Ooi and Wood, 2007; Jobe et al., 2012). The identification of the majority of the target genes repressed upon cell stimulation might ultimately also be useful in accounting for the differential effects of REST, such as its apparently variable role in cell death/survival, which has been observed in different experimental conditions.

The most exciting developments in the REST area are expected in the field of diseases. In the case of at least some psychiatric diseases, the most interesting finding will be a conclusive explanation of the mechanisms of REST action. At the moment, these mechanisms are conceived of only in general terms. In the case of neurologic diseases, additional studies, both *in vivo* and in various experimental conditions, are expected to carry out critical analysis of the various effects of moderate REST increases. Currently, the REST increases that occur in Alzheimer’s disease have been reported to be neuroprotective (Lu et al., 2014), while those in Huntington’s disease and epilepsy have been reported to contribute to the pathogenetic process ( Zuccato et al., 2007; Bithell et al., 2009; Buckley et al., 2010; McClelland et al., 2011a, 2014). The other developments we expect in the field involve therapies. Currently, the studies of valproic acid do not appear promising. In contrast, this might be the case for the drugs developed for therapies for Huntington’s disease. This and other approaches suggest that new pharmacological initiatives should be requested to rejuvenate the therapy for other brain diseases.

In conclusion, investigation of the roles and mechanisms of action of REST in adult neurons is still open, with at least potentially exciting avenues. Developments are expected to take place in both basic and translational fields. Possible pharmacological developments might ultimately have a considerable medical and social impact, which could be important, especially in aging populations. Further reviews in this area that are focused on at least some of the developments specified here, and include investigations using new experimental and technological approaches, could be needed in the next few years.

## Synthesis

The decision was a result of the Reviewing Editor Tallie Z. Baram and the peer reviewers coming together and discussing their recommendations until a consensus was reached. A fact-based synthesis statement explaining their decision and outliningwhat is needed to prepare a revision is listed below. The following reviewers agreed to reveal their identity: Douglas Coulter

Both referees found major improvement in the paper, and I concur. The organization and content are now in line with a state-of the art review on the timely topic of REST/NRSF.

The Reviewers had difficulty in fully following the review because of persisting in-optimal use of English. The recommendation is that the authors employ an Editor who is fluent an expert in scientific English. We shall be pleased to receive an edited manuscript.
